# Are Construction Managers from Mars and Workers from Venus? Exploring Differences in Construction Safety Perception of Two Key Field Stakeholders

**DOI:** 10.3390/ijerph19106172

**Published:** 2022-05-19

**Authors:** Mostafa Namian, Mohammadsoroush Tafazzoli, Ahmed Jalil Al-Bayati, Sharareh Kermanshachi

**Affiliations:** 1 Department of Construction Management, College of Engineering and Technology, East Carolina University, Greenville, NC 27858, USA; 2 School of Design and Construction, Voiland College of Engineering and Architecture, Washington State University, Pullman, WA 99164, USA; tommy.tafazzoli@wsu.edu; 3 Department of Civil and Architectural Engineering, Lawrence Technological University, Southfield, MI 48075, USA; aalbayati@ltu.edu; 4 Department of Civil Engineering, College of Engineering, University of Texas at Arlington, Arlington, TX 76019, USA; sharareh.kermanshachi@uta.edu

**Keywords:** safety perception, construction workers, construction managers, safety knowledge, safety culture and commitment, safety performance, safety support and communication, safety training, construction field

## Abstract

Persisting high rates of worksite accidents and injuries in construction projects indicate the urge to investigate the root causes and revisit safety practices in this industry. Consonance in perceptions and safety approaches has been identified as a fundamental factor in boosting projects’ safety. Discrepancies between how different elements of construction safety are perceived and handled by the key stakeholders, namely managers and workers, could be detrimental to worksite safety. This research studied how, if at all, the perception of four key construction safety components, including 33 sets of pairwise questions, is different in the lens of managers from workers. To explore safety perceptions, 133 construction professionals in the United States participated in the study and expressed their perceptions toward their own and counterparts’ (1) safety knowledge, (2) safety culture and commitment, (3) safety performance, and (4) safety support and communication. The results indicated that massive gaps in safety perceptions do exist between the construction managers and workers (26 out of 33 areas), and the magnitude varies for different safety elements. In all four categories, both managers and workers perceived a superior safety position for themselves and inferior for their counterparts. Further investigations revealed that the common ground between managers and workers is their consensus on proper communication and safety training as the key solutions to address such discrepancies. Construction safety professionals and practitioners can benefit from the results of this study to establish and implement strategies to foster communication and provide more effective safety training to bridge the existing gaps in the perception of safety by managers and workers.

## 1. Introduction

Accounting for one of the industries with high rates and numbers of work-related accidents [1], the construction industry is considered a hazardous workplace for workers. According to the United States Bureau of Labor Statistics, only in 2019, the occupational fatality in the US reached 5,333 workers, of which construction accounted for almost 20% or 1061 of the deaths [2]. The fatality rate for the construction industry was 10.2 per 100,000 full-time equivalent (FTE) workers in 2020, while for all workers in the country, it was 3.4 [3]. The alarming and constant high rates of work-related construction accidents and their dire consequences convey the urgent need for profound attempts to improve construction worksite safety.

In addition to the altruism aspect of construction safety, to avoid the massive direct and indirect costs of construction accidents, it is imperative to investigate how current safety practices can improve to achieve more effective measures to prevent accidents. To this end, a myriad of research has been conducted in different countries and various project types. Among them, the involvement of different stakeholders (i.e., managers and workers) in the safety management programs is a prominent factor [4,5,6,7]. Different stakeholders can have different perceptions when it comes to safety [8]. Although diverse perspectives offer benefits, different perceptions with huge gaps can create challenges in construction safety. Safety perception addresses the knowledge and awareness of construction players in identifying workplace hazards and their associated risks. This perception is validated when it stimulates a response to control the hazard and prevent the potential accident. In doing so, an individual who identifies the hazard and perceives the associated safety risks should have the knowledge and be equipped with the required tools and resources to adopt an appropriate response that can effectively mitigate or eliminate the risk [9,10].

Proper perception of safety risk depends on multiple factors that can break the error chain and prevent job site accidents [11]. Multiple tools and resources could be utilized in establishing and improving safety perception. For instance, periodic safety training, clarification of safety goals by the contractor, regular inspections, rigorous reporting, and insightful supervision can potentially improve the way hazards are identified and the associated risks are perceived by project personnel [12]. Effective safety perception can potentially contribute to reducing the costs associated with job site accidents [13,14], improving productivity [15], and positively impacting the contractor’s reputation [16].

Several studies have investigated the discrepancies and differences in perception of safety among different project stakeholders [17,18]. However, the perception of two key field players, namely managers and workers, has not been studied before. Although the significance of safety perception by the workers is known to many construction professionals [19], the possibility of workers’ different perspectives in perceiving hazardous situations can potentially make administrative safety practices less practical or effective. Dissimilar perceptions of the workers will also lead to responding differently from what project managers expect them [8]. A fundamental solution in filling the gap between project managers and workers in the way they decide to respond to safety risks is therefore understanding how each group perceives safety practices. In this study, the potentially different perception of project managers and workers of construction safety has been examined using a survey method. The findings of the research are expected to clarify the major divergences and enable project managers and safety practitioners to revisit their safety practices and administration policies by aligning both key stakeholders’ perceptions of safety.

## 2. Literature Review and Background

According to the Bureau of Labor Statistics [2,3], the rate of fatalities and injuries in the construction industry is five times higher than other industries’ average. Worksite accidents impose massive consequences on this industry, including severe injuries, permanent disabilities, and fatalities. Additionally, damages to project assets will, in turn, create substantial direct and indirect costs for workers, contractors, owners, and the economy. For instance, these incidents create substantial financial pressure on the industry to pay for workers’ compensation, insurance, liability, and legal prosecutions [20]. The results of a recent study revealed a connection between safety and productivity [15]. The authors of this study argued that productivity gains offset the costs required for establishing a safe working environment. In another study by Tam et al. [21], impairing contractors’ reputation was identified as the most adverse impact of site accidents. Additionally, in most cases, job site accidents will cause a delay and can damage the contractor’s reputation and reliability [22]. Table 1 shows some of the consequences of construction job site accidents [23,24]. 

A holistic approach to safety improvement in construction projects is administrating comprehensive safety practices to effectively detect potential hazards and the associated safety risks, provide immediate and appropriate preventive measures, as well as proper post-accident response to mitigate accidents’ impacts. This holistic system should include training, monitoring, and tracking as well as record keeping and accident data analysis [25]. According to the literature, such administration is primarily provided by human resources and subject to human errors [26].

Multiple approaches have been examined to enhance construction safety, and their impacts have been shown in the research focused on safety improvement practices in construction projects. Some of the major studied tools include worker’s training [25,27,28,29,30,31], effective communication [32,33,34], continuous documentation [4,35], proper use of equipment [36,37], close supervision [38], and advanced technologies [39] or combination of them [40]. Table 2 shows the main approaches studied in the research to improve safety (data from [41,42]).

Assessment of safety perception is commonly conducted through survey data collection. There is a plethora of research investigating this topic in various types of projects and different locations from the perspectives of different stakeholders [43,44,45]. However, the missing link in analyzing safety perception is investigating the inconsistency between major project players in their perceptions of safety components. The inconsistency can negatively affect establishing a safe environment and tailoring safety management programs based on the need of the beneficiaries. Such inconsistencies can create a gap between the definition of safety, goals to be achieved, and expectations to be fulfilled. In this study, it was examined if such a discrepancy exists and, if it does, in what aspects it is mainly manifested. The potential benefit of identifying these inconsistencies is identifying the aspects of safety perception that require to be revisited to gain consistency at a team level. The project players whose perceptions of safety components were compared in this study are construction managers and construction workers, the two key human assets of each project working hand-in-hand in the field [8]. 

To this end, the fundamental step is identifying the contributing and detrimental safety factors in a construction project. Examples of research conducted in this category are (1) studies focusing on accident theories, including Domino Theory [46], Hinze’s Distraction Theory [11,47], and human error theory [48]; (2) studies that apply models to investigate accidents’ causes such as the multiple causation model [49] and accident root cause tracing model [50]; (3) research on factors causing specific accident types, such as falls [51] and electrocution [52]; (4) the application of technology in detecting the causes such as image processing [53] and machine learning [54]; and (5) survey-based studies asking construction professionals [55].

**Figure 1:**
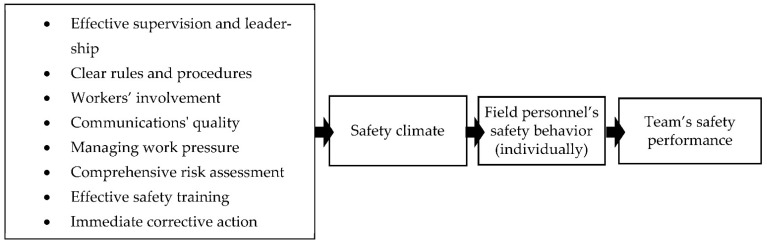
The link between safety measures and the team’s safety performance.

By tracking the root and origin of safety behavior, past research has identified multiple factors that can together form the safety climate and impact the safety performance of workers in any project [56]. Figure 1 shows some of these factors. As it is shown, the combination of multiple parameters can create various levels of commitment to safety in a typical construction work environment. There is consensus in the research about the strong connection between safety perception and safety climate and culture and the performance of workers [1,57,58]. Therefore, it can be inferred that safety perception could also be linked to the parameters listed in Figure 1. For example, differences in the received safety training, the level of work pressure that a worker or a manager feels, and the level of involvement in the safety programs shape their perception of safety and, therefore, their safety behavior which contributes to the safety performance of the project.

Safety perception has been identified in the research as a primary factor in creating appropriate responses as well as preventive measures to hazardous situations [59]. One challenge in perceiving safety is the involvement of different individuals, subgroups, departments, and divisions with different backgrounds, concerns, training, values, and cultures, to name a few. The diversity of perspectives leads to different interpretations and responses to situations [1]. While the diversity of safety perspectives is desired, different parties must be well aware of the fundamental differences that exist in their perceptions to properly tackle safety issues. Lack of such understanding leads to raising conflict among stakeholders rather than agreement to thrive implementation of effective safety management programs.

Another challenge of perceiving and determining risks on the job site is due to the inherited uncertainties that normally exist in construction projects. These uncertainties create perceptions and interpretations based on individuals’ insights. The insights, in turn, are influenced by multiple factors such as knowledge and experience that can vary among individuals [59]. This study aims to investigate if key construction safety elements are perceived differently among two key field players, construction managers and construction workers.

## 3. Research Method

It was mentioned that identifying the root contributors to safety in construction projects is a fundamental step in improving safety in this industry. It was also discussed that safety perception has been extensively identified in existing research as a pivotal factor in preventing job site accidents in construction. In this research, the safety perception was mainly reviewed from the perspectives of construction managers as well as construction workers. Each group of respondents was asked to answer a questionnaire targeted at assessing different criteria of safety perception. The questionnaire included paired questions in which one would self-assess the respondent’s performance, and the other one in the pair focused on the respondent’s assessment of the counterpart group’s performance in the same category. For example, the managers were asked to answer to what extent they agree that “I have sufficient safety knowledge”. In the next question, they were asked to express their opinion on a scale of zero (very strongly disagree) to 10 (very strongly agree) that “The construction workers have sufficient construction safety knowledge”. The idea was to investigate if there is a statistically significant difference between the two evaluations through a two-sample *t*-test. To identify the discrepancies between how managers and construction workers perceived safety, four main categories of (1) safety knowledge, (2) safety culture and commitment, (3) safety performance, and (4) safety support/communication were investigated. These criteria are explained in the following.

### 3.1. Safety Knowledge

**Figure 2:**
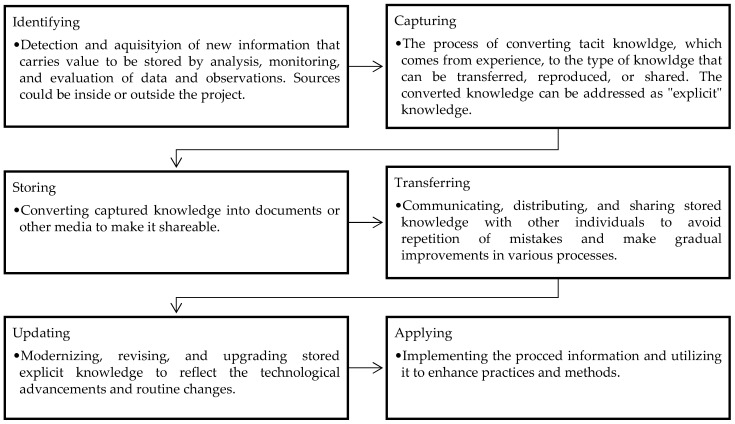
Essential steps for establishing safety knowledge management (adapted from [60]).

To enhance safety performance, safety knowledge and training are the prerequisites. Lack of sufficient safety knowledge leads to the inability of managers and workers to identify hazards in their workplaces and take corrective actions and prevent accidents [25]. Safety ignorance also leads to an increased possibility of unsafe behavior and violation of safety standards [10]. Hallowell [60] argued that the dynamic nature of the construction industry calls for the ongoing and updated establishment of knowledge [60]. The author identified four steps to manage this knowledge, as shown in Figure 2. According to this study, safety knowledge management is essential in enhancing safety. In this context, both the knowledge that is developed in individuals through their personal experiences, known as tacit knowledge, and codified documented knowledge, known as explicit knowledge, must be effectively utilized to transfer knowledge between individuals and groups [61]. 

As demonstrated in the figure, this structured process requires designing, administrating, and implementing multiple tasks in each step which indicates the necessity of establishing a knowledge management system. For instance, knowledge transfer requires a combination of information technology and human resource efforts [62,63,64] to manage, organize, and transfer tacit and explicit information. The significance of having a management system has been highlighted in the research [44,65,66]. Insufficient attempt in knowledge management leads to lost knowledge and failing its transfer to future projects [67]. Safety knowledge is a prerequisite for managers and workers to identify hazards and unsafe behaviors in their workplaces. Safety training programs are primarily developed to enhance the safety knowledge of construction employees [30], and as discussed earlier, providing effective and engaging safety training on a regular basis significantly helps to enhance the safety knowledge of managers and workers in construction.

In this category, a self-evaluation was conducted, plus an assessment in which the managers and construction workers evaluated the level of safety knowledge of the other stakeholder. In this study, the criteria that were assessed to evaluate safety knowledge are as follows: (1) having sufficient construction safety knowledge, (2) being able to identify safety hazards on the job site, (3) being able to recognize unsafe behavior of the construction workers or managers on the job site, (4) effective safety training programs provided to the construction workers, and (5) construction workers being engaged in the provided safety training.

### 3.2. Safety Culture and Commitment

In a study to identify root causes of construction accidents, Abdelhamid & Everett [68] identified three factors in the occurrence of construction accidents: (1) failing to identify hazardous situations prior to starting a construction activity, (2) ignoring a detected unsafe condition, and (3) unsafe behavior in the work environment. A work environment that promotes paying attention to safety mitigates all these three factors and leads to a reduction of the potential of accidents. Therefore, safety knowledge does not guarantee safe behaviors. For safety training and acquired safety knowledge to translate to tangible outcomes, safety culture and commitment must be promoted. Safety culture indicates the commitment to prioritize and value safety [69]. According to research findings, organizational factors are the major contributors to enhancing the safety climate in various industries, including construction [31,70,71]. The policies, procedures, and practices that are transferred to the workers, as well as the safety culture and safety commitment of the managers, could impact the safety climate and, therefore, the perception of safety by the workers [72].

Research in this area has identified multiple contributing factors in establishing the safety climate in an organization. The role of managers’ commitment to safety in developing the safety culture has been shown by multiple studies [73,74,75]. Another contributing factor to the establishment of the safety climate in an organization is peers’ behavioral patterns and the corresponding peer pressure. Based on this, the way workers identify hazards and perceive and evaluate the associated safety risks on the job site is impacted by their peer’s behavior [69,76]. Construction safety culture is defined as the policies and principles that guide safety decision-making, whereas construction safety climate is defined as the manifestation of these principles and policies within construction workplaces [31]. Safety culture is established and maintained by upper management and safety personnel, whereas safety climate is established through the safety culture and maintained by frontline supervisors and workers [31]. It is important to realize that safety culture should be recognized as a predictive indicator of safety climate and overall safety performance. In other words, safety culture indicates the commitment to prioritize and value safety [69].

In this study, the criteria that were assessed to evaluate safety culture and commitment are: (1) believing that safety is of paramount importance, (2) being dedicated, aligned, and accountable for safety-related issues, (3) not tolerating any unsafe behaviors on the job site, (4) generally caring about safety more than the other group (i.e., managers and workers), and (5) believing that safety is more of a priority than finishing tasks.

### 3.3. Safety Performance

Safety knowledge, in combination with the commitment to safety as a core cultural value, must lead to actions to perform safely and prevent accidents. Sjöberg et al. [77] defined risk perception as “the subjective assessment of the probability of a specified type of accident happening and how concerned people are with the consequences.” An example of this could be a foreman who detects a sick, exhausted, or sleepy worker who is in contact with hazardous tools or equipment. The foreman’s decision to intervene or ignore the exposure of workers to hazardous tools is highly impacted by his/her subjective assessment of the safety risk. Based on the research, people tend to underestimate the risk as they assume their experience or skills will be adequate to protect them against the potential hazard [10,78,79]. Impactful safety risk perception includes creating an in-depth understanding of the consequences of exposure to a hazard that could, in turn, motivate the worker to pause the current process of work and resolve the risk as the top priority.

A clear and precise understanding of the safety performance of managers and workers is integral in identifying a system’s weaknesses and fixing them. Based on the research, the majority of worksite accidents in the construction industry have roots in human errors [48,68,80]. Most human errors can be eliminated, or their risk can be significantly mitigated by implementing safety measures, establishing a culture of commitment to safety, and putting it a priority for all workers. The prerequisite of creating such an environment, however, will depend on the extent to which construction personnel are aware of possible safety measures and their ability to connect their knowledge to the work environment by implementing their safety knowledge on the field as the priority backed by safety culture and commitment. In other words, each construction personnel must have a realistic evaluation of his/her safety performance to be able to reinforce the strengths and address the weaknesses. Implementing safety training knowledge in daily practices on the job sites is an indicator of the safety performance of construction workers [9]. Following safety rules and standards is also another indicator of safety performance in construction [30].

In this study, the criteria that were assessed to evaluate safety performance are the following: (1) using the learned provided safety training materials at daily work, (2) following safety rules and practices on the job site, (3) having an acceptable safety performance, (4) having regular safety meetings held on the job site, and (5) believing that the participants are responsible for preventing construction accidents.

### 3.4. Safety Support and Communication

Safety support and communication catalyze safety enhancement efforts. The impact of effective communication in preventing safety hazards and collaborative prevention of potential injuries has been shown in countless studies [81,82]. Studies have also shown poor communication leading to job site accidents as a widespread problem [83]. On the other hand, facilitating safety communication helps workers to express their safety needs and concerns to be addressed by managers. Different technologies and methods such as toolbox talks [33] and pre-task safety planning [4] have been shown to positively impact job site safety through fostering safety support and communication. Safety managers and practitioners must clearly communicate their safety expectations and involve workers in the safety decision-making process [74,84]. Providing feedback in a timely manner to workers based on their safety performance has also been shown to have a substantial positive impact on construction safety [10].

In this study, the criteria that were assessed to evaluate safety support and communication are the following: (1) explicitly communicating safety commitments and support to construction workers, (2) workers having a concise understanding of their managers’ safety expectations, (3) providing formal and informal safety feedback frequently enough to workers, (4) workers being open to the provided safety feedback by managers, (5) considering enough safety incentives to strengthen the construction worker’s safety performance, and (6) assessing to what extent workers communicate their safety needs and concerns.

## 4. Data Analysis and Results

A total of 80 construction managers and 53 construction workers participated in the study. The respondents were randomly selected from the construction professionals working in Alabama, Arizona, California, Colorado, Florida, Georgia, Kansas, Louisiana, Missouri, New Jersey, New York, North Carolina, Ohio, Philadelphia, South Carolina, Tennessee, and Virginia. To evaluate the safety perception of managers and workers, the participants responded to 33 pairwise questions. Out of 4389 (133 × 33) collected answers, 55 questions remained unanswered. The missing information was substituted with the group average [85]. 

Table 3 shows the demographic information of the two groups of survey respondents. The roles of the managers included branch manager, civil and structural engineer, construction coordinator, construction manager, construction safety director, construction supervisor, field engineer, foreman, lead engineer, manager of project controls, office engineer, operation manager, owner, senior construction manager, site assistant manager, substation design manager, vice president, general contractor, and superintendent. The roles of the workers included carpenter, concrete finisher, electrician, field technician, helper, ironworker, journeyman, machine operator, plumber, rehabilitation specialist, tradesman, and mason. The types of projects included commercial, industrial, maintenance, rehabilitation, renovation, and infrastructural projects.

### 4.1. Self-Assessment for Safety Perception

In the first step, the analysis focused on self-evaluation of safety perception by the construction managers as well as the workers. As detailed previously, the questions were provided in four categories of safety knowledge, safety culture and commitment, safety performance, and safety support/communication. Using the 11-point Likert scale, the respondents were asked to evaluate themselves in these categories by a score ranging from zero (“very strongly disagree”) to 10 (“very strongly agree”). Table 4 shows the average of both managers’ and workers’ responses to the self-assessment statements within the four categories.

**Figure 3:**
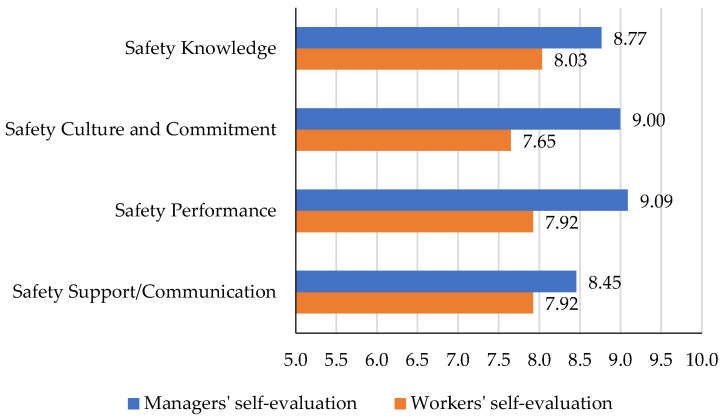
Comparing the self-evaluation of the two groups in four categories of safety perception.

To compare the construction managers’ and workers’ self-evaluations, the bar chart in Figure 3 is shown. As it can be seen in all categories, managers’ self-evaluations surpass that of workers. The results showed that safety support and communication is the category of safety perception that has the highest potential for improvement among the four categories for managers. From the workers’ perspectives, however, the safety culture and commitment category has the lowest score, which indicates its highest potential for improvement. The most extreme difference is seen in safety culture and commitment evaluation and the lowest difference is seen in safety support/communication, which is one of the lowest-rated categories indicating that both managers and workers are struggling with challenges in this area.

### 4.2. Assessment of the Counterpart Group for Safety Perception

As part of the study, the managers were asked to assess construction workers and, on the other side, the construction workers were asked to assess the managers in the same four categories of safety knowledge, safety culture and commitment, safety performance, and safety support/communication. The 11-point Likert scale was similarly provided to capture the responses. Table 5 shows the results.

**Figure 4:**
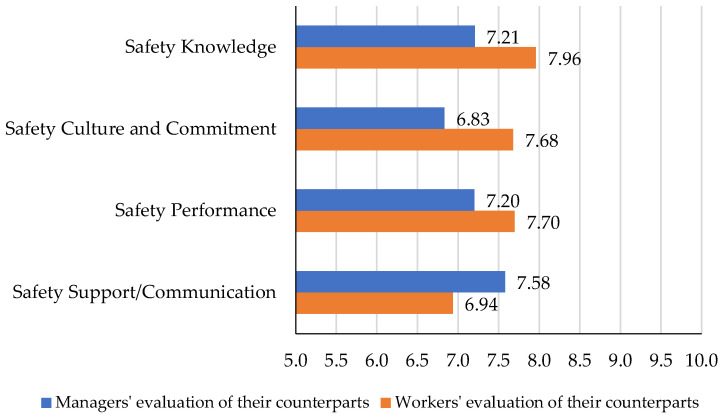
Comparing the opposite groups’ evaluations in four categories of safety perception.

The results of construction managers and workers evaluating the perception of safety by their counterparts’ groups could be compared to identify the most and least critical categories. The results are shown in Figure 4. It can be inferred from the bar chart that managers identified construction workers’ safety support and communication as their strengths, followed by safety performance and safety knowledge. The lowest average score among the four criteria of safety perception has been given by the managers to the workers in the category of safety culture and commitment, which shows a critical need for improvement.

The construction workers, on the other hand, ranked managers’ safety knowledge, safety performance, and safety culture and commitment as their strengths. The workers gave the lowest score, among the four categories, to managers’ safety support and communication, which indicates the most critical factor that the managers need to improve.

### 4.3. Comparing Self-Assessment of Construction Managers with the Workers’ Assessment

**Figure 5:**
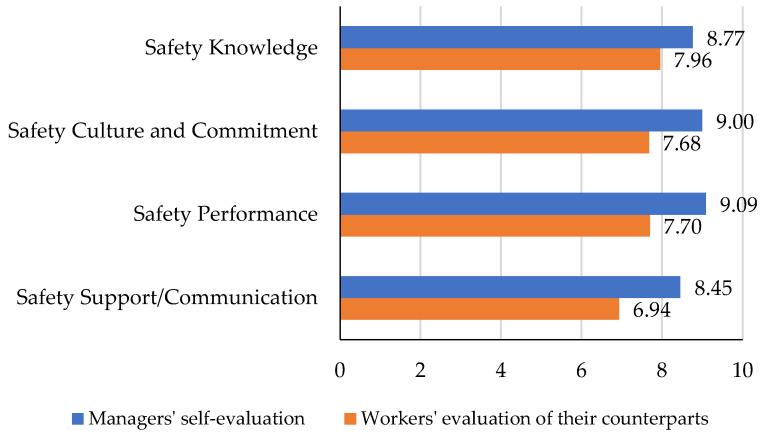
Managers’ safety evaluation.

**Figure 6:**
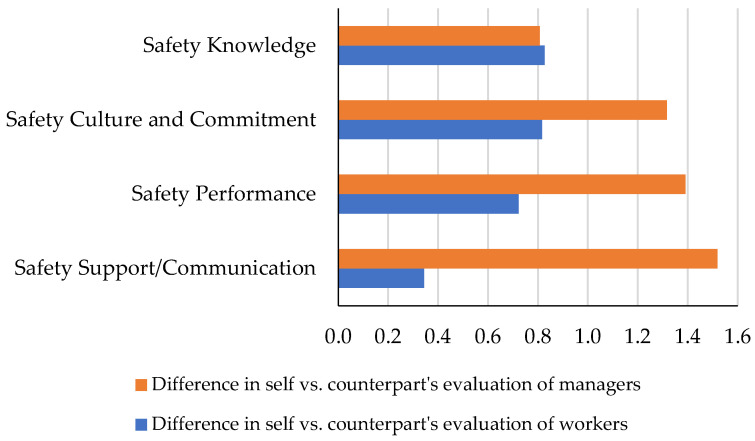
Differences in self vs. counterparts’ evaluation of managers and workers.

In this part, it was investigated if a gap could be noticed between the way construction managers assess themselves and the way workers evaluate them. To do so, the evaluation results in each of the four categories of safety perception were compared. The results are shown in Table 6, Figure 5, and Figure 6. Statistical analysis was conducted to see if the difference between the two evaluations was significant. The null hypothesis was that based on the results, with 95% confidence, there was no statistically significant difference between the two evaluations. A two-sample *t*-test was conducted, and the *p*-values showed that in all the four categories, the self-evaluations differ from workers’ evaluations of managers. These results can show that managers’ interpretation of their safety perception is different from how the workers assess them.

### 4.4. Comparing Self-Assessment of Construction Workers with Managers’ Assessment

**Figure 7:**
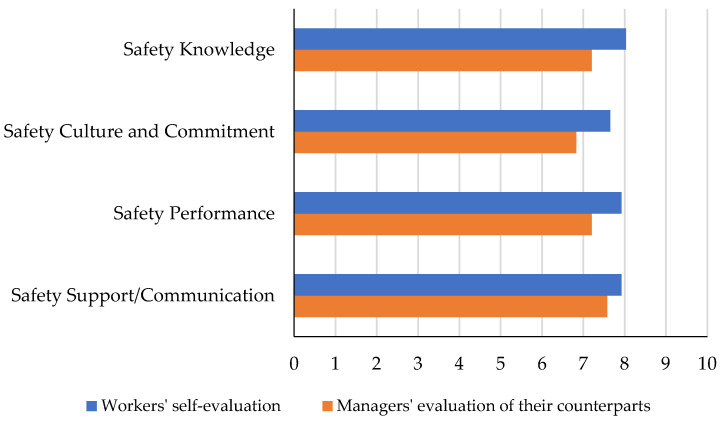
Workers’ safety evaluation.

The possible gap between the two assessments, that is, the workers’ assessment of themselves and how the managers assess them, was investigated. To check if the observed gap indicates a statistically significant difference, a *t*-test was performed on the data. The null hypothesis was set as no statistical difference between the scores at a 95% confidence level. The results of the tests are provided in Figure 6 and Figure 7, and Table 7. According to the findings, in the two categories of safety Knowledge and safety culture and commitment, the *p*-value rejected the null hypothesis and validated the difference between the two assessments. In the meantime, test results showed that managers’ assessment of workers and their self-assessment in the two categories of safety performance and safety support/communication are not statistically different.

### 4.5. Discrepancy among Construction Managers and Workers in Perception of Safety

In addition to the examination of four groups of safety perception, the analysis of each pair of questions further revealed that massive gaps in the perception of safety among managers and workers do exist (see Table 8). The results of the two-sample *t*-tests corresponding to each pair of questions are discussed in the following.

As can be seen in the following table, there are statically significant differences in the perception of managers and workers in 26 out of 33 pairs of comparisons (*p*-value < 0.05). Regarding safety knowledge, workers admit that their managers have a higher level of safety knowledge (S1). Their managers asserted that their workers’ safety knowledge is considerably lower than their own knowledge. However, both groups’ self-evaluations scored higher than the evaluation of their peers in the other group, and the differences were statistically significant. Concerning the ability to identify safety hazards (S2) and recognize unsafe behavior (S3), both groups of managers and workers expressed that they are able to better identify job-site hazards compared to their counterparts. Managers think the training that they provide their workers is effective (S4), yet their workers are not highly engaged (S5). This assertion is contradictory since high engagement is an essential aspect of effective safety training [25], and workers should not be solely blamed. On the other hand, it is more reasonable to coincide with workers’ opinions that the provided safety training is not effective enough to engage them.

Huge disparities exist in valuing safety (S6) and being dedicated to the related issues (S7) by managers and workers from the perspectives of their counterparts. These two statements are among the top three elements with the highest level of disagreement between managers and workers. However, both managers and workers agree that managers are less likely to tolerate the unsafe behavior of workers in construction projects (S8), although a statistically significant difference still exists in evaluating managers’ tolerance of unsafe behavior. Managers claim that they have a superior safety attitude to their peer workers, and workers implicitly confirm that in their responses to the survey (S9). The most extreme gap in perception of construction safety is reflected in prioritizing safety over getting the job done (S10). Both sides accuse the other side of sacrificing safety in dilemmas to finish a task in a shorter time. This raises serious concerns regarding a substantially critical and regular safety decision-making situation that almost all construction projects experience. This requires immediate actions to be taken to reconcile the perception of managers and workers to ensure the safety of workers and projects is not compromised to complete the tasks.

Managers and workers, unfortunately, disclose that not all safety training materials are used in practice (S11). This is in alignment with the past research that discovered typical safety training outcomes are largely undermined and not translated to tangible results when workers return to the field [9]. In a similar pattern, it was seen that managers think they follow safety standards better than workers (S12), and workers think their managers violate safety standards more often than themselves. Both sides of managers and workers evaluate their overall safety performance more positively than their opposite sides (S13). Another gigantic disagreement arose when the respondents were asked about holding regular safety meetings (S14). Generally, workers believe that not enough regular safety meetings are held, while managers disagree. This can be explained by the fact that, unfortunately, workers are not involved in all safety meetings and safety decision-making, despite the proven positive contribution of workers’ safety involvement to construction safety enhancement [9]. Managers and workers’ responses revealed that they both consider managers to feel more responsible for preventing construction accidents (S15).

Managers reported that they explicitly communicate their safety commitment and support (S16), and the workers are well aware of their safety expectations (S17). However, workers believe managers must more explicitly demonstrate their safety commitment and support, and there are some barriers to a concise understanding of the safety expectations of their managers. Managers and workers have an agreement on providing formal and informal safety feedback by managers to their workers, although there is some room for improvement (S18). Workers clearly emphasized that they are open to the provided feedback (S19); however, managers have a different opinion which does not indicate workers welcome the safety feedback. Generally speaking, the workers score significantly lower than the managers concerning safety incentives (S20). Workers believe more safety incentives must be provided by their managers to recognize their safe behavior. Another vast area of disagreement emerged when managers’ openness to workers’ safety requests and feedback was asked (S21). Managers believe they are utterly open to their workers’ safety requests, while workers have serious concerns regarding easily communicating their safety needs (S22). It seems the perception of workers more realistically show the safety communication level in construction workplaces considering both sides’ responses of evaluating how often workers communicate their safety needs and concerns. Both managers and workers agree that such communications do not happen on a regular basis (S23).

## 5. Post Hoc Investigation of Managers’ and Workers’ Safety Perception

The managers and workers were asked open-ended questions to further investigate why such a massive gap in their safety perceptions exists. As expected, most managers and workers analyzed the problem from different perspectives. A manager believed the difference is normal and mentioned, “Everyone has a different perception in reality. Managers look at safety more closely because they want to ensure the workers are properly protected. Workers are more task orientated, or job focused”. Similarly, a young superintendent with two years of experience reasoned this difference stems from differences in their responsibilities and said, “Managers look at the entire project and what needs to be done whereas workers only look at trying to complete certain tasks”. A construction coordinator added the problem to be “Conflict between practical and theoretical [approaches of managers and workers]”. One of the participants detailed that “Workers are often the first to point out hazards while managers are often more knowledgeable about the specific rule/codes”. A superintendent cited the language barrier as a contributing factor. Moreover, an electrician with 20 years of experience saw this as a generation-gap problem and said, “I think the lapses in generation have something to do with it as there are guys who worked in the industry that had zero safety standards compared to new guys knowing nothing but regulations. The common ground is filled with those who just let them hash it out”. 

A considerable portion of both managers and workers blamed each other without trying to put themselves in their peers’ shoes. Among managers, for example, a 56-year-old site superintendent with 40 years of experience criticized workers because “The workers want to do what is easy. Being safe is not always easy”. An assistant project manager who worked in construction for 10 years argued workers’ lack of proper safety risk perception is the root cause and articulated that “The workers do not get penalized for unsafe practices as long as they don’t get hurt”. In addition, a project manager (28 years old with 5 years of experience) claimed, “Workers don’t care about safety because they don’t think anything will happen to them, and if something does, they think our company will pay them for injuries”. Another manager said workers sometimes misconduct in the name of safety and said, “Because workers sometimes use safety to get out of doing work”. A project manager who worked in the industry for 40 years said, “Workers view safety practices as a hardship and typically feel it slows down work progress. Managers view safety as a necessary aspect of wellbeing”. Another manager thought, “The differences are the workers think that managers are unfair when they are just doing their work to make sure everyone is safe”. Similarly, various workers blamed managers for being the source of the problem. For instance, a Hispanic concrete finisher with 30 years of experience said, “All managers care about is getting the job done,” or another worker added, “Time taken away for safety is [the] time taken away from getting the job done. This appears to be how managers view things”. A 33-year-old worker with eight years of experience blamed managers that they do not properly communicate their commitment and explained, “Managers need to do things other than just issuing memos and giving speeches if they expect the workers to believe their safety commitment”. A young carpenter’s apprentice condemned managers’ approach and mentioned, “Managers are only about cost and schedule and don’t care if we are working ourselves to death”.

An assistant project manager (39 years old with 12 years of experience) admits that sometimes they compromise workers’ safety as mentioned “As managers, our responsibility is people, time, and money. Sometimes we make time and money our focus instead of people.” A 46-year-old superintendent of a commercial project with extensive years of experience (22 years) criticized both sides and said, “I believe workers think managers prioritize lawsuit avoidance over worker safety, and managers don’t believe workers can understand safety rules and regulations.” Another participant also held both sides accountable for the situation and expressed that “Workers feel that management is only worried about the bottom line [i.e., profit]. It is ok to take shortcuts to save a dollar. Managers give the impression that time is of the essence.” 

Many managers and workers pointed out the long-debated balance between productivity-safety as the source of discrepancies. A duct hanger who worked on a commercial project with 22 years of experience mentioned, “We view some safety [practices] as slowing them down. Managers do not understand how much following all safety rules can slow them down”. A 46-year-old carpenter with 28 years of experience pictured the dilemma as a constant battle of safety and productivity and said, “Construction projects are always behind and will always want to be faster, which makes safety difficult”. In addition, it has been argued that the structure of payment nudges workers to perceive safety differently because “A lot of workers get paid based on the amount of work or number of jobs completed; therefore, the workers don’t have the same mindset as the managers”. A 50-year-old project manager who had 24 years of experience explained, “Most of the subs on my job site are paid by the job. Therefore, their goal is to get the job done as soon as possible. I would like for the job to get done quickly as well, but without compromising safety”.

Several managers and workers concurrently indicated the lack of communication as the leading cause of discrepancies in their safety perception. A manager admitted that “A difference comes from lack of communication on the job site” and a worker claimed “The managers don’t tell us their point of view enough”. Several factors contribute to the communication problem. For example, workers are afraid of the potential consequences as a manager revealed that “Sometimes the workers are scared to say anything in fear of getting fired”. A worker demanded managers change their communication approach and explained, “If they started caring about us instead of dollar signs and calendars, we would be in a much better spot. All they have to do is talk to us like people and have some form of care and devotion to making sure we are taken care of”. A carpenter highlighted an important yet often neglected safety challenge which is inconsistency in safety culture and practices among different companies. He explained that “We work on different job sites through the week sometimes. Some of our superintendents enforce hardhats and safety glasses as a minimum, but some don’t. We get a little confused going from one job site to the next when it comes to safety”. In fact, this worker explained how there is a lot of inconsistency in the enforcement of safety rules from job to job. This causes some workers to take an unsafe approach to work on construction sites. When they see their immediate supervisor not following safety rules, they feel that they are not mandated to follow the rules.

Many workers mentioned that lack of field experience among managers contributes to the gap in perception of safety. For example, a worker asserted that “The managers don’t really know what is going on in the field”. A 21-year-old worker who started construction when he was 18 explained, “From my experience, the managers don’t always get out into the job site nearly as much as the everyday boots on the ground workers. Therefore, they can’t fully understand all the hazards on the job”. A related note was from a 31-year-old machine operator with 10 years of experience that did not perceive differences in safety perception and declared that “The perception is the same. My superintendent used to operate heavy machinery and knows how important safety is”. Contrarywise, a 58-year-old worker who worked in construction as a carpenter for 40 years in response to the possibility of reconciling the disagreements mentioned disappointedly that “Never have been, probably never will be—two different lungs on the social ladder- do what you’re told or will get someone who will!” 

Although a few participants (10.4%) were disappointed to reconcile the discrepancies, the majority of managers and workers were optimistic (76.4%). A considerable number of participants (13.2%) argued that reconciliation is contingent on circumstances such as long-term commitments, trust of workers in their managers, transparency, consistency, mutual respect, and avoiding “us vs. them” mentality. Among the solutions that the participants mentioned to address the massive gap in the safety perception of managers and workers, “communication” (*n* = 23) and “safety training” (*n* = 11) were among the most frequently suggested answers. A 27-year-old project engineer responded that “Yes, I believe it we maintain open lines of communication and set the expectation early in a job and hold each other accountable—we can achieve a mutual understanding. It is important to work with each other and not police, seek to understand the differences, and take the time to make sure a worker understands the safety concerns and OSHA standards”. Another participant simply outlined the solution to be “more communication on reasonable expectation”. A manager explained the novel perspective of proper communication based on the accumulated experience: “In my experience, most workers are able to recognize most safety issues but often do not react because they feel that they do not have the authority”. This statement implicitly encourages managers to give authorities to workers to take control actions to achieve higher safety outcomes. In addition, effective yet comprehensive safety training can help to curb the problem, as explained by a manager: “As a general note, often the GC [General Contractor] spends money on training for safety but has no required safety training for subcontractors in their related trades. What happens is the GC’s superintendent is expected to provide safety to a bunch of employees of subcontractors who are neither trained for safety or care about it”. Another response outlined that “Yes [it is reconcilable] if the companies would be investing more time on training and OSHA would remove the monetary factors from safety training”. Such opinions are promising that despite the massive discrepancies among managers and workers in the perception of construction safety, reconciliation is achievable.

## 6. Summary and Conclusions

This study investigated the difference between how four key aspects of safety are perceived on construction job sites by managers versus workers. The urge to improve safety in the construction industry was discussed by comparing the high rates of accidents in this industry with other industries. The integral role of safety perception in creating safe behavior and safe performance was also explained. In this research effort, safety perception was assessed in four main categories, including (1) safety knowledge, (2) safety culture and commitment, (3) safety performance, and (4) safety support and communication. 

To answer the research question, a survey was conducted among construction professionals in two groups of managers and workers to evaluate themselves as well as their counterpart peers in their understanding, commitment, and performance in each of the four mentioned categories. The categories were broken down into sub-criteria for a more detailed assessment. The results of the data analysis conveyed a massive gap between the assessment of the two groups on their safety perception versus how the other group perceives. 

Based on the statistical analysis of each category, the category with the lowest score indicated the highest potential for improvement. The findings show that in the viewpoint of the construction managers, their “safety support and communication” needs more improvement than other measured categories. The workers had a slightly more realistic perception; however, they identified “safety culture and commitment” as the category that requires more determination for improvement. The findings also suggest that the strengths and weaknesses of each of the two studied groups assessed by themselves vary significantly from how the counterpart group evaluates them. 

Among 33 sets of pairwise questions, two-sample *t*-tests revealed discrepancies in responses of managers and workers in 26 pairs of questions related to the perception of safety. “Prioritizing safety over getting the job done”, “believing that safety is of paramount importance”, and “[being] dedicated and accountable for safety-related issues” are the top three areas with the highest level of disagreement between managers and workers. On the other hand, managers and workers, to some extent, agreed on (1) workers’ [relatively low] level of engagement in safety training programs, (2) managers’ approach to dealing with unsafe behavior of workers, (3) relatively low level of utilizing training materials in the field by workers, (4) managers’ responsibility to prevent accidents, (5) managers providing formal and informal safety feedback to workers, and (6) workers insufficiently communicating their safety needs and concerns. These results could shed light on the paths for boosting safety programs and how the effective improvement measures for construction managers differ from those of construction workers. Finally, the findings of the post hoc investigation discovered “communication” and “safety training” as potential solutions to tackle the problem by professionals and practitioners.

Despite its merits and benefits to the construction industry, the study has a few limitations that must be considered. First, safety perception in this study was limited to 33 questions. However, the perception of safety in construction is more complicated, and further aspects can be considered. Second, the managerial hierarchy in different companies is different, which can impact the safety perception of managers. This important factor was not considered in this study. Lastly, more managers participated in this study than workers. However, in reality, for a typical construction project, the number of workers exceeds the number of managers by far.

## Figures and Tables

**Table 1:** Consequences and costs of construction job site accidents.

Project Party	Costs
Owner	Additional project costs resulting from delayed delivery Costs of replacing contractor, project manager, or other parties selected by the owner Possible need to reapply for project permits
Worker (Injured worker)	Loss of future earnings Non-reimbursable medical treatment costs Costs of rehabilitation
Contractor	Replacing injured workforces and associated costs of training and recruiting Loss of productivity due to interruption in operations Legal expenses Maintaining resources resulting from delayed delivery Reduce profitability of the project Negative impacts on the firm’s reputation Fines and penalties (particularly when the public and/or their properties are involved)
Economy and Society	Insurance Medical treatment Compensations Direct and indirect costs of conducting legal processes Delayed utilization of the facility under construction Indirect costs of investigation and inspection Loss of human capital Medical subsidy costs

**Table 2:** Major safety improvement approaches.

Approach	Concept/Definition	Examples
Personnel Selection	Based on the common belief that a small percentage of employees cause the majority of accidents	- Setting criteria and screening methods to select personnel who are committed to the safety - Using individuals’ characteristics to predict their safety behavior
Advanced technologies	Utilizing existing and emerging technologies to improve safety on the job site	- Detecting hazardous situations through image processing - Construction automation to reduce the human’s role in risky situations
Behavioral methods	Providing training that teaches safe behavior followed by observation	- Offering high-engaging safety training to workers - Setting safety goals and other incentives - Providing feedback for worker’s safety-related behavior
Signages	Using sufficient, if not abundant, safety signs and posters as constant reminders	- Signs notifying a potential hazard - Signs reminding mandatory use of PPE - Signs motivating safe behavior in multiple spots
Assigning Safety Champions	Assigning a leader in each team or crew to focus on commitment to safety among the team members	- Assigning the foreman/superintendent in each crew as the safety champion
Stop Work Culture/Power	Granting authority to workers of all ranks to stop work when they are exposed to work under hazardous conditions with insufficient safety protection	- A worker can refuse to perform the work orders by the foreman/superintendent for safety concerns
Quality Circle	Sharing experiences and solving issues together through regular meetings	- One team shares their experience of how they successfully dealt with a safety concern - One team asks for suggestions about an upcoming safety challenge they are facing

**Table 3:** The demographics of survey respondents.

Group	Minimum Age (Years)	Maximum Age (Years)	Average Age (Years)	Minimum Experience (Years)	Maximum Experience (Years)	Average Experience (Years)
Managers	21	64	44	0.33	42	19
Workers	18	58	34	0.5	40	12

**Table 4:** Results of self-evaluation of safety perception for construction managers and workers.

Assessment Category	Statement	Managers’ Self-Evaluation	Workers’ Self-Evaluation
Safety Knowledge	I have sufficient construction safety knowledge.	8.82	7.94
	I am able to identify safety hazards in the job sites.	8.97	8.53
	I am able to recognize the unsafe behaviors of the construction workers/managers and superintendents on the job site.	9.09	8.58
	I/we have provided effective safety training programs to the construction workers.	8.18	NA
	Attending the safety training programs, I was engaged in the provided material.	NA	7.08
Safety Culture and Commitment	I believe safety is of paramount importance.	9.67	8.94
	I believe I am dedicated, aligned, and accountable for safety-related issues.	9.29	8.57
	I do not tolerate any unsafe behaviors of [NA/my peer] * construction workers on the job site.	9.30	7.49
	Generally, I care more about safety than the [workers/managers] *.	7.88	5.70
	I believe safety is more of a priority than finishing tasks.	8.83	7.55
Safety Performance	I use the learned provided safety training material in my daily work.	NA	7.21
	I follow safety rules and practices on the job site.	9.44	8.17
	My safety performance is acceptable.	9.16	8.47
	I/we hold regular safety meetings on the job site for the workers.	8.64	NA
	I believe I am responsible for preventing construction accidents.	9.12	7.85
Safety Support/Communication	I explicitly communicate my safety commitment and support to the construction workers.	8.59	NA
	I have a concise understanding of my manager/superintendents’ safety expectations.	NA	7.77
	Formal and informal safety feedback is provided frequently by me to the workers.	7.54	NA
	I consider enough safety incentives to strengthen the construction workers’ safety performance.	7.57	NA
	I am open to the provided safety feedback [by the managers].	NA	8.79
	I am open to safety feedback/requests from the construction workers.	9.41	NA
	The construction workers can easily communicate their safety needs and concerns to me.	9.25	NA
	I always communicate my safety needs and concerns to the managers/superintendents.	NA	7.21

* The first option was shown to the group of managers, and the second was shown to the group of workers.

**Table 5:** Results of evaluation of the counterpart group for safety perception.

Assessment Category	Statement	Managers Evaluating Workers	Workers Evaluating Managers
Safety Knowledge	The [construction workers/managers and superintendents] * have sufficient construction safety knowledge.	7.16	8.17
	The [construction workers/managers and superintendents] * are able to identify safety hazards in the job sites.	7.36	8.21
	The [construction workers/managers and superintendents] * are able to recognize the unsafe behaviors of the construction workers/managers/superintendents on the job site.	7.42	8.19
	Effective safety training programs have been provided to me at the project.	NA	7.10
	The construction workers attending safety training programs are engaged in the provided material.	6.90	NA
Safety Culture and Commitment	The [construction workers/managers and superintendents] * believe safety is of paramount importance.	7.43	8.21
	I believe the [construction workers/managers and superintendents] * are dedicated, aligned, and accountable for safety-related issues.	7.24	7.68
	The [construction workers/managers and superintendents] * do not tolerate any unsafe behaviors of the construction workers on the job site.	6.68	7.77
	The [construction workers/managers and superintendents] * believe safety is more of a priority than finishing tasks.	5.97	7.06
Safety Performance	The construction workers use the learned provided safety training material in daily work.	6.95	NA
	The [construction workers/managers and superintendents] * follow safety rules and practices on the job sites.	7.12	7.89
	The [construction workers’/managers’ and superintendents’] * safety performance is acceptable.	7.33	7.98
	Regular safety meetings are held on the job that I work [by managers].	NA	6.98
	The [construction workers/managers and superintendents] * believe they are responsible for preventing construction accidents.	7.38	7.94
Safety Support/Communication	The construction workers have a concise understanding of my safety expectations.	8.48	NA
	The managers/superintendents explicitly communicate their safety commitment and support to me and other construction workers.	NA	7.12
	Formal and informal safety feedback is provided by my managers/superintendents to me and other construction workers.	NA	6.77
	The construction workers are open to the provided safety feedback.	7.41	NA
	Enough safety incentives are considered by the managers/superintendents to strengthen the construction workers’ safety performance.	NA	5.73
	The managers/superintendents are open to my safety feedback/requests.	NA	7.48
	We [the construction workers] CAN easily communicate our safety needs and concerns.	NA	7.58
	The construction workers DO communicate their safety needs and concerns to me.	6.85	NA

* The first option was shown to the group of managers, and the second was shown to the group of workers.

**Table 6:** Results of comparing managers’ self-assessment with workers’ assessment of managers.

Comparison Category	Results of the *t*-Tests (*p*-Value)	Are the Results Statistically Significant?
Safety Knowledge	0.023	Yes
Safety Culture and Commitment	0.001	Yes
Safety Performance	0.001	Yes
Safety Support/Communication	0.021	Yes

**Table 7:** Results of comparing workers’ self-assessment with managers’ assessment of workers.

Comparison Category	Results of the *t*-Tests (*p*-Value)	Are the Results Statistically Significant?
Safety Knowledge	0.0410	Yes
Safety Culture and Commitment	0.0156	Yes
Safety Performance	0.3068	No
Safety Support/Communication	0.1560	No

**Table 8:** Results of comparing managers’ and workers’ self-assessments with their corresponding counterparts.

#	Topic/Statement	Safety Evaluation of Managers				Safety Evaluation of Workers			
		Managers’ Perspective	Workers’ Perspective	Δ	*p*-Value	Managers’ Perspective	Workers’ Perspective	Δ	*p*-Value
Safety Knowledge									
S1	Having sufficient construction safety knowledge	MQ1 = 8.82	WQ2 = 8.21	0.61	0.021 *	MQ2 = 7.16	WQ1 = 7.94	−0.79	0.013 *
S2	Ability to identify safety hazards on job sites	MQ3 = 8.97	WQ4 = 8.25	0.72	0.010 *	MQ4 = 7.36	WQ3 = 8.53	−1.17	0.000 *
S3	Ability to recognize unsafe behavior of workers/managers	MQ5 = 9.09	WQ6 = 8.23	0.87	0.002 *	MQ6 = 7.42	WQ5 = 8.58	−1.17	0.000 *
S4	Providing effective safety training	MQ7 = 8.18	WQ7 = 7.15	1.03	0.011 *	-	-	-	-
S5	Engaging in provided safety training	-	-	-	-	MQ8 = 6.90	WQ8 = 7.08	−0.18	0.683
Safety Culture and Commitment									
S6	Believing that safety is of paramount importance	MQ9 = 9.67	WQ10 = 8.21	1.46	<0.0001 *	MQ10 = 7.43	WQ9 = 8.94	−1.51	<0.0001 *
S7	Dedicated and accountable for safety-related issues	MQ11 = 9.29	WQ12 = 7.68	1.61	<0.0001 *	MQ12 = 7.24	WQ11 = 8.57	−1.33	0.000 *
S8	Not tolerating any unsafe behavior of workers	MQ13 = 9.30	WQ14 = 7.77	1.53	<0.0001 *	MQ14 = 6.68	WQ13 = 7.49	−0.81	0.065
S9	Superior safety attitude compared to the counterpart	MQ15 = 7.88	-	-	-	-	WQ15 = 5.70	-	-
S10	Prioritizing safety over getting the job done	MQ16 = 8.83	WQ17 = 7.06	1.77	<0.0001 *	MQ17= 5.97	WQ16 = 7.55	−1.57	0.000 *
Safety Performance									
S11	Practicing safety training material in daily work	-	-	-	-	MQ18 = 6.99	WQ18 = 7.21	−0.22	0.602
S12	Following safety standards on the job sites	MQ19 = 9.44	WQ20 = 7.89	1.56	<0.0001 *	MQ20 = 7.12	WQ19 = 8.17	−1.05	0.004 *
S13	Overall safety performance is acceptable	MQ21 = 9.16	WQ22 = 7.98	1.18	<0.0001 *	MQ22 = 7.33	WQ21 = 8.47	−1.14	0.001 *
S14	Regular safety meetings are held on the jobsite	MQ23 = 8.64	WQ23 = 6.98	1.66	0.001 *	-	-	-	-
S15	Feeling responsible to prevent construction accidents	MQ24 = 9.12	WQ25 = 7.94	1.17	<0.0001 *	MQ25 = 7.38	WQ24 = 7.85	−0.47	0.250
Safety Support/ Communication									
S16	Managers explicitly communicate their safety commitment and support	MQ26 = 8.59	WQ26 = 7.12	1.48	0.000 *	-	-	-	-
S17	Workers concisely understand managers’ safety expectations	-	-	-	-	MQ27 = 8.48	WQ27 = 7.77	0.71	0.039 *
S18	Providing formal/informal safety feedback to workers	MQ28 = 7.54	WQ28 = 6.77	0.67	0.105	-	-	-	-
S19	Workers’ openness to the provided safety feedback	-	-	-	-	MQ29 = 7.41	WQ29 = 8.79	−1.38	0.000 *
S20	Providing enough safety incentives to workers	MQ30 = 7.48	WQ30 = 5.73	1.74	0.000 *	-	-	-	-
S21	Managers’ openness to workers’ safety requests/feedback	MQ31 = 9.41	WQ31 = 7.48	1.93	<0.0001 *	-	-	-	-
S22	Workers can easily communicate their safety needs and concerns	MQ32 = 9.25	WQ32 = 7.58	1.67	<0.0001 *	-	-	-	-
S23	Workers always communicate their safety needs and concerns	-	-	-	-	MQ33 = 6.85	WQ33 = 7.21	−0.36	0.426

* *p*-Value < 0.05.

## Data Availability

The generated data for this study will be available upon reasonable request to the corresponding author.
